# The double-edged sword of short-form video platform use: Chinese undergraduates’ perceived academic consequences and self-regulatory behaviours on Douyin

**DOI:** 10.3389/fpsyg.2026.1863958

**Published:** 2026-10-26

**Authors:** Ziqi Jia

**Affiliations:** School of Marxism Studies, Nanjing University of Science and Technology, Nanjing, Jiangsu, China

**Keywords:** Chinese undergraduate students, cross-sectional survey, Douyin, perceived academic impact, self-regulatory behaviour, short-form video

## Abstract

**Background:**

Short-form video platforms such as Douyin/TikTok have become pervasive among college students, yet participants’ reported perceptions regarding their learning-related consequences remain paradoxical. While offering flexible, accessible learning resources, these platforms may also pose challenges for students’ self-regulatory behaviours.

**Methods:**

A cross-sectional survey was conducted with 268 undergraduate students from three different types of higher education institutions in China. Outcomes were measured through students’ self-reported perceived academic impact rather than objective academic performance measures. Usage patterns, perceived academic impact, self-regulatory behaviour and perceived value and actual use of Douyin use were analyzed using descriptive statistics and comparisons.

**Results:**

Over two-thirds of participants (67.17%, *n* = 268) reported using Douyin for academic purposes, acknowledging benefits such as improved knowledge access and learning engagement. However, 39.17% reported having ambivalent effects and 20.9% reported habitual, unconscious use. There was a notable descriptive discrepancy between perceived educational value and actual educational use: while students recognized the drawbacks to focus and productivity, many had difficulty controlling usage. In other words, higher-level participants in our sample showed more adaptive self-regulatory behaviours (no formal statistical tests were performed).

**Conclusion:**

Short-form video platforms represent a double-edged sword in undergraduates’ self-reported learning experience. They offer valuable microlearning opportunities but also challenge students’ self-regulatory behaviours in the case of poor self-control. These results provide empirical reference for future studies of short-video learning experiences in undergraduates.

## 1 Introduction

Short-form video platforms have become hugely popular globally among higher education students in recent years (Zhang, 2025; Chen, 2025). Although the user penetration data shows widespread adoption in many countries, the effects of perceived learning differ significantly among regions (Gong and Tao, 2024). Douyin (TikTok, a Chinese short-form video application) is the most popular application in China. Its algorithm-driven content recommendation system reshaped how students access information, entertain themselves, and engage in informal learning (Shutsko, 2020). Unlike traditional social media, which is text-based interactions, Douyin’s 15–60 s vertical video format provides highly stimulating and personalized content streams leveraging cognitive reward mechanisms, stimulating both immersive engagement and concerns about distractions (Wang and Zhao, 2025; Zhou, 2023). In China, higher education institutions continue to promote students’ digital literacy cultivation in recent years. This anonymous questionnaire-based research strictly follows China’s Personal Information Protection Law; no personally identifiable private information was collected from participants. This article is also relevant to Sustainable Development Goal 4 (SDG 4: Quality Education). Understanding undergraduate’s self-reported experiences with short-form digital media can provide empirical reference for higher education institutions to develop digital literacy education and support inclusive quality education for university students.

The academic implications of such platforms remain unclear and uncertain. One is that Douyin provides microlearning opportunities, which allow students to access condensed professional knowledge, examination preparation materials and skill tutorials in fragmented yet accessible format. User-generated content and algorithmic personalization support self-directed learning and just-in-time information acquisition. On the other hand, increasing evidence suggests that the “addictive design” of short-form video platforms (with infinite scrolling, variable reward schedules and instant gratification) may impede sustained attention, aggravate the cognitive-load and fragment study routines essential for academic success (Zhang and Huang, 2022; Lin, 2023).

This paradoxical nature makes Douyin a “double-edged sword” in higher education contexts where the same technology which facilitates educational access can, according to perceptions, also challenge learning depth and self-regulatory capacity. Recent meta-analysis confirms a low-to-moderate negative correlation between the multitasking of academic media and academic success (Shi, 2023), but these statistics obscure the senses of students who use these platforms every day. Understanding how undergraduates’ perceive, interpret and navigate the academic consequences of using Douyin is critical to developing evidence-based interventions and pedagogical strategies that harness its potential while mitigating cognitive costs.

Existing studies on university students’ short-video use also cover other Asian and ASEAN contexts (He and Yang, 2025; Li and Li, 2021). Regional differences in digital culture and higher education systems suggest that context-specific studies should be conducted on Chinese students. Despite extensive research on social media’s effects on academic outcomes, significant gaps remain in the literature on students’ subjective self-reported perceptions of social media effects, especially in China. Existing studies use mostly quantitative objective outcomes measures, often without taking into account the self-reported experiences of students when they surf online these platforms daily (Wei, 2022; Jonas et al., 2024). Comparing effects for the two institutions are also not available. Comparisons between university resources, academic culture, and student demographics are limited to understanding how perceived effects are moderated.

We used survey data collected from 268 students in three different universities (a national “Double First-Class” research university, a provincial Normal University and a municipal technology institute) to assess Chinese students’ perceptions of how their Douyin experience affects their academic experiences. We addressed these gaps by three questions: RQ1: What are Chinese undergraduates’ self-reported usage patterns of Douyin for academic and recreational purposes? RQ2: What are undergraduates’ perceived academic impacts associated with Douyin use across students from different institutional backgrounds? RQ3: What self-reported reasons and perceived contributing factors correspond to positive and negative perceived academic impacts of Douyin engagement?

## 2 Theoretical framework and literature review

### 2.1 Theoretical framework

Understanding what students think about the consequences of using the platform has to include the technology acceptance model with cognitive and motivation models. Davis’s (1989) technology acceptance model posits perceived usefulness (PU) and perceived ease of use (PEU) as the motivations to use the platform. Recent meta-analyses of TAM in educational contexts show that perceived usefulness predicts learning related motivations, while ease-of-use-to-usefulness pathways differ depending on context (Scherer et al., 2019; Shibchurn and Yan, 2015). In Douyin, TAM can help us understand why students might use the platform freely if they believe tangible learning benefits outweigh the cost of interaction.

However, TAM’s focus on intentional adoption decisions does not capture the compulsive habituated usage behavior of short-form video platforms. Social Cognitive Theory (Bandura, 1986) emphasizes reciprocal determinism between environmental stimuli, personal cognition, and behavior. In this framework, self-efficacy beliefs about digital self-regulation and expectations about outcome of course consequences play an important role in usage behavior. Zhao et al. (2021) show that the method facilitates learners’ engagement and meta-cognition, thus encouraging social construction of knowledge. We use the Technology Acceptance Model (TAM) as background interpretive framework. We do not adopt validated TAM measures and thus cannot test TAM related hypotheses.

Cognitive Load Theory was first proposed by Sweller (1988). It distinguishes intrinsic cognitive-load (inherent to learning tasks), extraneous load (inherent to poor instructional design) and germane load (inferred from schema construction). For a review of the theory see Gauvain (2010). Short-form video platforms may also impose extraneous cognitive load (in the form of constant attention switching, notification interruptions and “switch costs” for multitasking) (Kirschner and De Bruyckere, 2017). Tang et al. (2025) explored how to improve the intuitiveness of teaching while maintaining mathematical rigor by integrating dynamic visualization techniques such as GeoGebra for facilitating students transition from intuitive cognition to formal understanding. Visualization-based instruction significantly reduces the cognitive load of abstract concepts, and deep understanding of mathematical concepts is promoted. This consistent, though modest, effect suggests that perceived distraction may reflect real-world cognitive-resource depletion. Cognitive Load Theory distinguishes intrinsic, extraneous and germane cognitive load. Our questionnaire does not measure these cognitive-load dimensions. Any inference about cognitive-resource depletion derived from usage-time statistics remains speculative and serves only for post-hoc interpretation.

Finally, Distraction Conflict Theory (Baron, 1986) provides a theoretical framework for understanding how digital media-generated attentional conflict may affect academic performance. According to this framework, environment distractions cause conflict in mind by forcing attentional division between competing tasks, which results in decreased processing depth and learning efficiency. Distraction Conflict Theory can provide an interpretation of attention disturbance caused by digital content. We do not have validated scales to measure attentional-conflict constructs for this survey; therefore, we cannot empirically verify its proposed mechanism.

We take Social Cognitive Theory (Bandura, 1986) as the main theoretical framework to interpret interactions between environment, personal cognition and self-regulatory behaviours. Technology Acceptance Model (TAM), Cognitive Load Theory and Distraction Conflict Theory are only used as interpretive frameworks in discussion. No measured scales for the main constructs of these additional theories have been included in our questionnaire; hence we cannot test their hypotheses empirically in this survey.

### 2.2 Literature review

#### 2.2.1 Short-form-video use in higher education

The global proliferation of short-form video platforms has changed digital consumption behaviours of university-aged students (Shutsko, 2020; Zhou, 2023). As the most popular domestic player, Douyin (TikTok) uses algorithm driven recommendation to provide highly stimulating and personalized vertical short-video content. Previous cross-country surveys showed that young adults often use short-video applications for entertainment, information seeking, and social interactions (Montag et al., 2021). In the literature, short-video applications are widely used on universities in Western and Asian settings. However, most large descriptive studies focus on general usage prevalence rather than learning-oriented activities among undergraduates.

Existing studies have confirmed the widespread popularity of short-form video among college students, yet they provide limited targeted descriptions distinguishing entertainment-driven use from academic-oriented use. This study supplements this gap by mapping both recreational and academic usage patterns among Chinese undergraduates.

#### 2.2.2 Educational use of short-video platforms

Short-form video platforms also provide opportunities for informal microlearning. Condensed video clips can provide fragmented knowledge, skill tutorials and examination preparation resources, thus reducing the barriers to just-in-time knowledge acquisition (Zhao, 2024). Some intervention-oriented studies implemented TikTok into university courses and proved its potential to increase learning engagement and career awareness (López-Carril et al., 2025). Self-media short-videos have been shown to create authentic language learning scenarios for college level foreign language education (He and Jin, 2019). Educational functions are often secondary to entertainment functions on commercial short-video platforms. User generated content also faces uneven quality and fragmented knowledge systems.

Prior intervention research has verified short-videos’ educational potential under structured teaching arrangements. Less is known about students’ spontaneous, unguided educational use outside formal curricula. This paper focuses on voluntary out-of-class platform engagement to enrich understanding of informal micro-learning reality.

#### 2.2.3 Self-regulatory behaviours in digital-media context

Digital media impose high demands on users’ self-management. Social cognitive theory shows reciprocal interactions between environment cues, individual cognition and behaviour (Bandura, 1986). In digital media the individual self-regulatory behaviour determines whether users filter information and avoid mindless scrolling. Existing work shows that infinite scroll interface and variable reward algorithm design trigger habitual checking behaviours and limit sustained concentration (Zhang and Huang, 2022; Kirschner and De Bruyckere, 2017). Most of the experimental work uses experimental settings or general sample. Few descriptive surveys target undergraduates concrete self-regulatory behaviours when using short video applications.

Theoretical frameworks have clarified the importance of self-regulation in digital contexts, but empirical descriptions of undergraduates’ real-world self-regulatory behaviours during spontaneous Douyin use remain insufficient. This study collects self-reported behavioral indicators to fill this descriptive gap.

#### 2.2.4 Perceived versus objective academic-related outcomes

A large amount of literature examines associations between short-video and academic outcomes. Some studies report negative association between short-video and objective academic outcomes (Wang and Tian, 2025; Zhang et al., 2023); others argue platform effects are context dependent; short-video can provide educational benefits when it is embedded in formal teaching (Cheng, 2025; Gong and Tao, 2024). However, most quantitative studies use objective measures such as grade point average, assignment scores or experimental cognitive test results. Subjective student perceptions (how learners decide whether short videos have positive or negative consequences) are neglected. Subjective perceptions shape student learning and self-management decisions even when they are not objective records (Abdullah and Ward, 2016; Michikyan et al., 2015).

Prior literature prioritizes objective academic outcome indicators, while students’ subjective perceived academic impacts receive comparatively little empirical attention. This study centres participants’ self-reported perceptions to complement outcome-oriented objective-metric-based research.

#### 2.2.5 Discrepancy between perceived value and actual use

The difference between people’s positive attitude towards a technology and their real behaviours has been a longstanding issue in social science research. The Technology Acceptance Model explains users’ adoption intention based on perceived usefulness and perceived ease of use (Davis, 1989). Positive perceived usefulness does not translate to consistent practical use. For educational technology situations, learners may recognize learning value of a tool but not use it consistently because of distraction or habit-driven operations (Sheeran and Webb, 2016). Existing short-video studies rarely compare students’ perceived educational value of short-video resources and their actual frequency of learning-oriented platforms.

Few short-video-focused empirical works examine the mismatch between students recognized educational value of platform content and their real-world learning-oriented usage frequency. This paper documents such descriptive discrepancies among sampled Chinese undergraduates.

#### 2.2.6 Institutional-context differences

University institutional background influences student digital media practice. Different admission competitiveness, curriculum requirements, campus digital literacy resources and peer atmosphere may produce different student technology use patterns (Lau, 2017; Baumgartner et al., 2017). Students from high-selection institutions have different academic pressure and resource conditions than students from municipal teaching-oriented universities. Most short-video and learning studies pool University participants together, whereas descriptive evidence between different institutions is scarce in Chinese higher education settings.

There is limited empirical evidence describing how undergraduates from different-tier Chinese higher-education institutions differ in short-video usage and related perceived academic impacts. This study draws samples from three institutions with different admission tiers to provide cross-institutional descriptive evidence.

By foregrounding student voice in a technology-saturated educational landscape, this research provides empirical reference for future work concerning critical digital literacy and sustainable technology practices. The paradigm of this study is shown in Figure 1.

**Figure 1:**
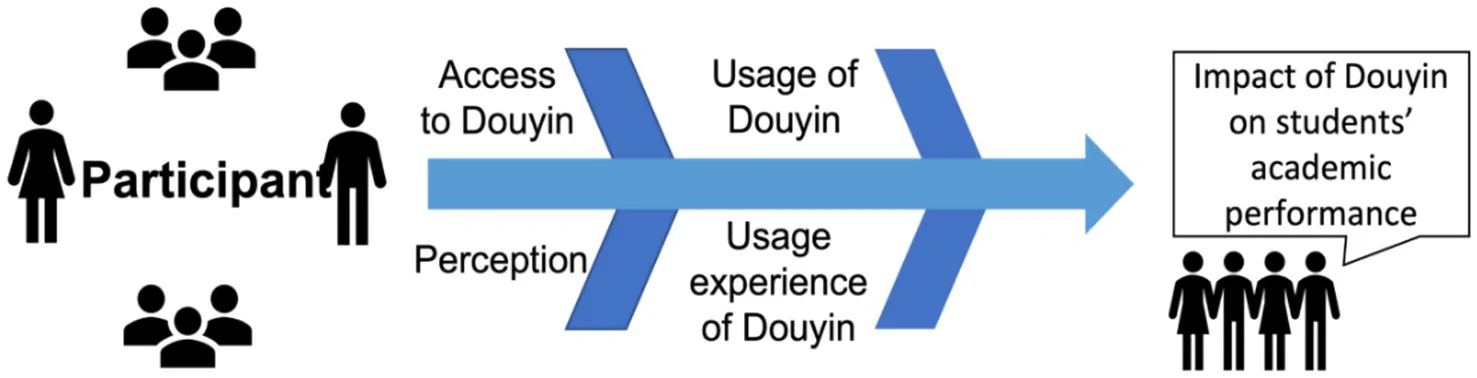
Paradigm of this study.

## 3 Methods

### 3.1 Research design and objectives

We used a cross-sectional survey to study Chinese university students’ experiences of the Douyin use. We used a quantitative cross-sectional self-administered questionnaire to study Chinese university students’ experiences with Douyin use. We did not employ qualitative or inductive data analysis. Questionnaire data were collected using the Wenjuanxing (Questionnaire Star) online survey platform. All responses were anonymous. No personal information (Mobile Phone Number or WeChat ID) was retained. Only the team had access to cleaned data.

### 3.2 Instrumentation and data collection

Primarily, data were collected using self-assessed digital questionnaires deployed during summer semester. Three higher education institutions with different admission levels were chosen for this work. Individual students were recruited via snowball convenience sampling among WeChat groups and QQ groups of these universities. This is not probability based stratified sampling. Our sample cannot be generalized to all Chinese undergraduate students and sampling bias may exist. External validity is limited to participants from these three Xuzhou universities.

Eligibility criteria required participants to be (a) currently enrolled undergraduates, (b) 18+ years old, and (c) active Douyin users. We excluded identifiable information for anonymity. Before starting, we briefed participants on the objectives, rights to participate, and withdrawal procedures without penalty. Average completion time was 15 min.

The survey instrument was constructed and distributed through Wenjuanxing (Questionnaire Star), a widely used Chinese academic survey platform. Snowball sampling was performed by QR codes and hyperlink sharing on course specific WeChat and QQ group chats, ubiquitous communication channels on Chinese campuses. The aim was to reach across disciplines while using existing social networks for recruiting. We collected 279 questionnaire responses. Exclusion step 1: 11 participants who said they did not use Douyin were excluded. No duplicate submissions or incomplete questionnaires were found. Attention check items were not included in the questionnaire; this limitation will be noted in limitation section. Final valid analytical sample N = 268. Given descriptive research focus, we did not perform any inferential testing. Since we did not perform any inferential testing, no power analysis was performed. Final valid responses provide sufficient sample size for descriptive frequency statistics and cross-tabulation for the three sampled institutions.

The questionnaire was self-designed by the research team. It covers participant demographic information, Douyin usage behaviour, self-reported perceived educational impacts, self-regulatory related behaviours and reported reasons for perceived outcomes. Items have single choice, multiple-choice and Likert type responses scales. Two educational technology experts reviewed the draft questionnaire for content validity before it was released. A small-scale test was performed to improve item wording. Since no off-the-shelf validated multiitem latent scales were used for most constructs in this survey, Cronbach’s alpha reliability coefficients were not calculated. This limitation is acknowledged in limitation section, and this study does not use a validated multidimensional self-regulated-learning scale. We only collected several single-item indicators of concrete self-regulatory-related behaviours (e.g., unconscious habitual checking of short-video applications). This study focuses on these observable behavioural reports rather than measuring the full latent construct of self-regulated learning; no standardized validated SRL instrument was deployed.

### 3.3 Analytical strategy

All analyses used descriptive statistical techniques (frequency counts, percentages, crosstabulations). No inferential statistical hypothesis test (chisquare test, regression test) was performed. The observed subgroup differences are descriptive patterns of this sample and not statistically verified population differences.

The analytical work went through the following steps: (1) descriptive profiling of Douyin engagement behavior; (2) bivariate analysis of attitude differences according to university tier and user; (3) thematic regularities in perceived academic impact; and (4) theoretical interpretation of emergent patterns. These descriptive analysis procedures are carried out to ensure empirical results are based on participant reports while conversing with existing theories (TAM, Cognitive Load Theory and Distraction Conflict Theory).

## 4 Results

### 4.1 The popularity of Douyin

Before presenting the empirical findings, this section outlines the sample’s demographic and institutional characteristics. Data collection yielded 279 responses from undergraduates matriculating at three Xuzhou-based higher education institutions: China University of Mining and Technology (CUMT), Jiangsu Normal University (JNU), and Xuzhou University of Technology (XUT). After subtracting 11 students who did not use Douyin, the sample contained 268 valid cases, representing different levels of Chinese higher education (CUMT, JNU, Municipal second) and different Gaokao admission thresholds.

Platform engagement: 67.17% (*n* = 268) of participants indicated using Douyin for academic purposes, while 32.83% (*n* = 268) denied using it. Cross-institutional distribution: CUMT (*n* = 83, 31.0%), JNU (*n* = 79, 29.5%) and XUT (*n* = 106, 39.6%) (Figure 2). This proportional distribution suggests relatively uniform platform penetration across institutions, even if XUT is modestly overrepresented. Academic progression: First-year students dominate with 60.07% (*n* = 161) followed by sophomores [27.62% (*n* = 74), juniors 4.85% (*n* = 13)] and seniors (7.46%, *n* = 20). This inverse relationship between cohort seniority and Douyin engagement suggests less dependence on platform as students’ progress through their degree program.

**Figure 2:**
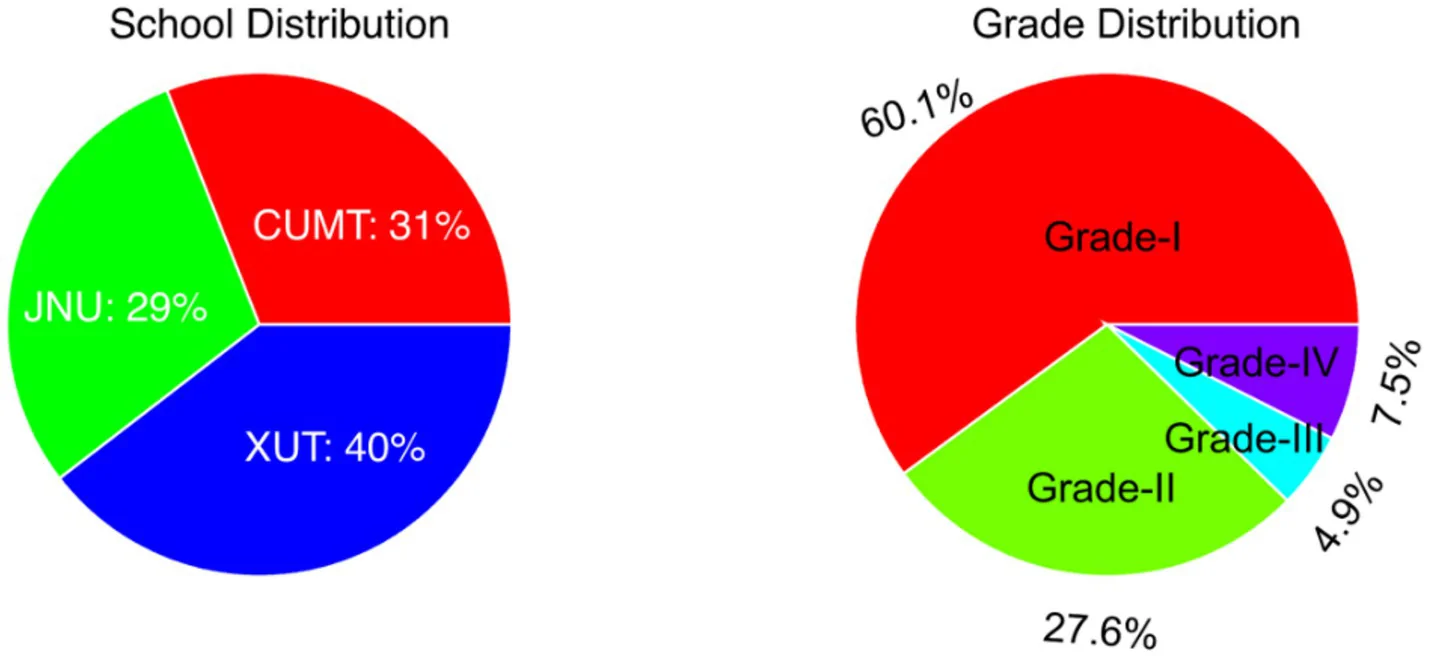
The school and grade distributions for Douyin usage among undergraduate students (total valid sample *n* = 268).

These results support that Douyin has become deeply embedded in Chinese undergraduates’ daily lives, but the content types driving this engagement are still interesting to study. Genre preferences of the respondents (Figure 3), shows that humor was the most popular (55.69%, *n* = 268), followed by lifestyle documentation/vlogging (55.49%, *n* = 268) and skill-acquisition tutorials (45.66%, *n* = 268) (Figure 3). Educational content ranked third. Diversion is still the main motivation, but instrumental learning is a major secondary use case.

**Figure 3:**
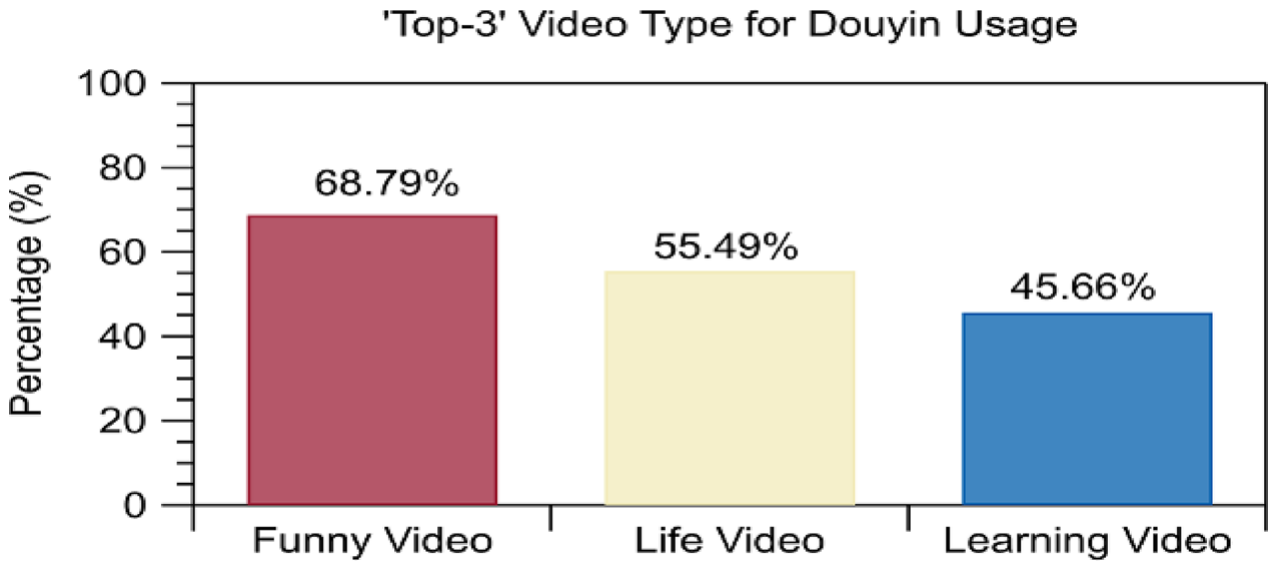
Top-3 video types for Douyin usage among the Chinese undergraduates (*n* = 268). Multiple-response questions were applied for this survey item; percentages sum to over 100%.

However, institutional stratification differs considerably. For example, when separating student content preferences across three universities, students at the more selective institution (CUMT) preferred to rank educational videos second, while JNU and XUT chose to rank videos third and fourth, respectively. In other words, students at the more selective institution (CUMT) were more likely to use the platform for academic purposes, possibly reflecting differences in self-regulation, academic socialization or curriculum demands shaping how students at elite institutions deal with -short-form video consumption.

### 4.2 Views about Douyin’s impact on academic performance

The previous analysis shows that Douyin is both a recreational platform and educational tool among Chinese undergraduate students. In this section, we discuss how students perceive the effects of Douyin on their study. Most students (72.76%, *n* = 268) have some sense of the impact of Douyin on their study and 27.24% (*n* = 268) said short videos did not influence their study.

For instance, 18.66% (*n* = 268) reported predominantly positive perceived effects (mostly moderate rather than transformative), and 14.93% (*n* = 268) reported negative perceived effects. Most strikingly, 39.17% (*n* = 268) reported ambivalent evaluations, both positive and negative. This tripartite distribution—in which ambivalent evaluations outweigh purely positive or purely negative assessments—underscores the complex pedagogical profile of this platform. Instead of producing fixed scholastic outcomes, Douyin-related perceived impacts appear context-dependent; net perceived effects are contingent on individual usage patterns, consumed content, and self-regulatory behaviours.

Cross-institutional variation in impact valence is pronounced (Figure 4). XUT respondents exhibited the strongest optimism, with 54% (*n* = 106) endorsing beneficial academic effects—substantially exceeding CUMT (28%, *n* = 83) and JNU (18%, *n* = 79). Conversely, negative appraisals concentrated disproportionately at JNU (45%, *n* = 79), followed by CUMT (32.5%, *n* = 83) and XUT (22.5%, *n* = 106). We observe descriptive differences in perceived impacts of the academics between three institutions (Figure 4). Interpretations of self-regulatory capacity, academic socialization and academic pressure are considered as tentative hypotheses for future work and are not tested directly in this dataset.

**Figure 4:**
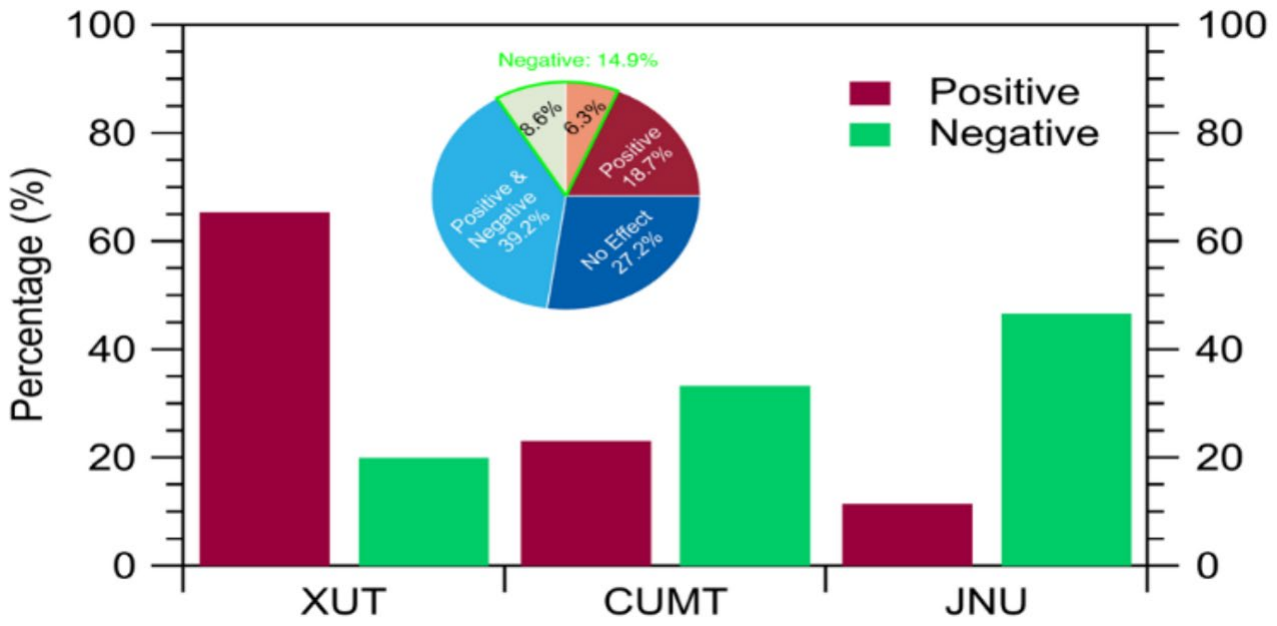
Perceived academic impacts of Douyin use by valence (pie chart, *n* = 268) and institution (bar chart; CUMT *n* = 83, JNU *n* = 79, XUT *n* = 106).

### 4.3 Self-reported reasons linked to perceived academic-related outcomes of Douyin engagement

After having established both the widespread nature of Douyin engagement and students’ differing views of its consequences, this section examines the perception of contributing factors driving these perceptions. Figure 5 summarizes the main attributional beneficial self-reported factors. Instrumental gains focus on domain-specific expertise and timely access to information (e.g., graduate entrance examination materials, civil service test updates), supported by 56.65% and 45.09%, respectively, (Figure 5). Other facilitators include peer-instructor connectivity, motivational enhancement and work-rest modulation.

**Figure 5:**
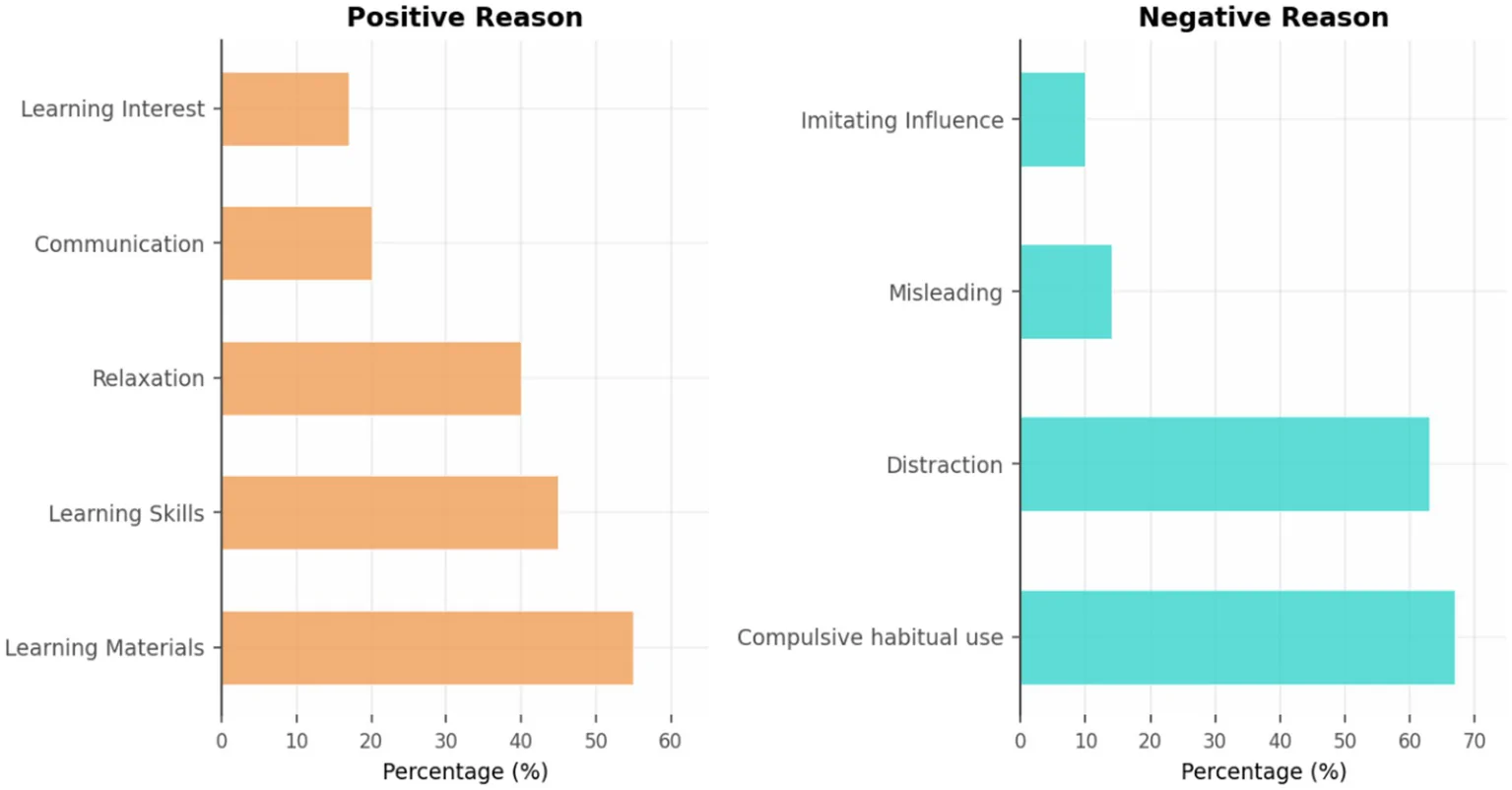
Percentage distribution of students’ numbers by positive and negative reasons.

Self-reported problematic usage-related patterns predominate among negative attributions, specifically compulsive consumption displacing study hours (temporal displacement) and attentional fragmentation during learning episodes (cognitive interference). A minority cited commercial livestreaming emulation as a competing demand on scholarly time.

The above attributional factors are supported by behavioral indicators that show how engagement intensity and functional deployment shape perceived results. Figure 6 shows time distributions. Usage Duration. Daily consumption distribution is positively distributed: 43.28% (*n* = 268) reported under one hour, 29.48% (*n* = 268) reported one hour to two hours, 15.67% (*n* = 268) reported two hours to three hours and 11.57% (*n* = 268) reported high volume daily usage duration over three hours. This only describes self-reported consumption time and should not be considered as a diagnosis of addiction or problematic use disorder. Thus, most participants consume moderately, but a small minority reported daily short-video consumption. Although 45.66% of participants cited skill-acquisition as one of their motivations for using Douyin, instrumental learning-oriented use remains sporadic: 58.96% (*n* = 268) use Douyin as a learning tool only occasionally, 31.72% (*n* = 268) never do so, and only 9.32% (*n* = 268) report frequent educational engagement. This suggests that the potential of learning videos is not clear. Perceived Efficacy: 47% (*n* = 268) acknowledge the power of learning videos and 36.6% (*n* = 268) report no clear benefit and 16.4% (*n* = 268) dismiss pedagogical value altogether. Generally positive efficacy appraisals combined with restrained instrumental use suggest that perceived usefulness does not translate into sustained learning.

**Figure 6:**
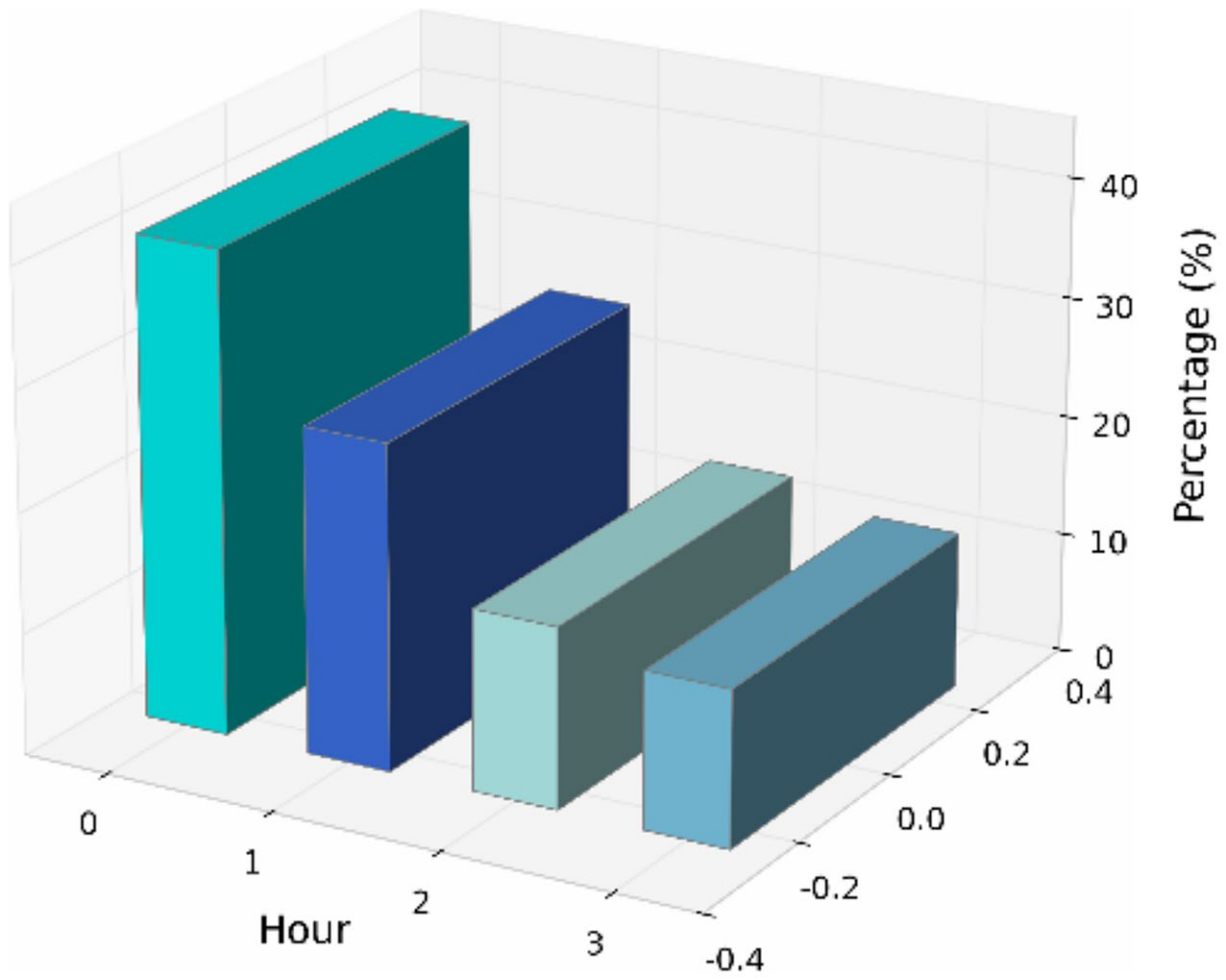
Students’ number percentage distribution based on the time for the usage of Douyin (*n* = 268).

This underutilization coincides with a large automaticity of usage. When asked about non-deliberate usage during study episodes, 79.1% reported rare (33%) or sporadic (46.1%) spontaneous use, and 20.9% (*n* = 268) habitual unconscious use (Figure 7), exceeding the proportion of participants reporting high-volume daily usage over 3 h. This separation between intentional pedagogical use and reflexive consumption suggests that platform architecture may develop habitual checking behaviors independent of conscious learning goals.

**Figure 7:**
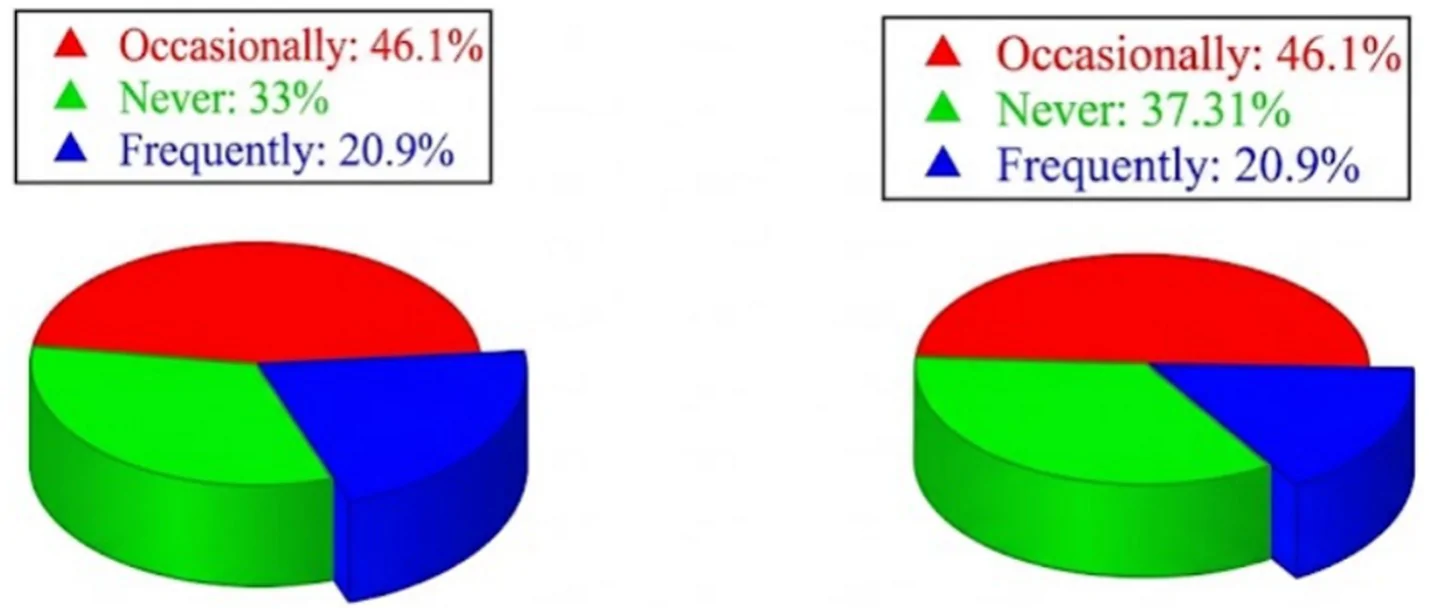
Percentage of students’ number distribution based on the frequency of open Douyin (*n* = 268).

To isolate specific disruption pathways, we asked the respondents to weigh the impact of their scholastic interference. The majority (87.3%) said Douyin rarely (37.31%) or occasionally (49.99%) interferes with their schedule (only 12.7%) interferes with their plan regularly (*n* = 268). This is similar to unconscious habitual access among participants, suggesting disruption is more confined to high-risk behaviors than spreading across samples.

Following this experiential mapping, continuance intention was assessed among those reporting negative impacts. Strikingly, persistence versus discontinuation split evenly, indicating that perceived harm alone is insufficient to predict behavioral termination—possibly reflecting subjective preference, optimism bias, or limited alternative coping strategies among participants.

## 5 Discussion

This section summarizes the descriptive patterns observed for our sample of Chinese undergraduate students at 3 Xuzhou based institutions. Interpretations made based on external published work are marked as speculations since many of the constructs were not measured in the current cross-sectional questionnaire. These observations are only based on our sample and cannot be generalized to the whole sample of Chinese undergraduate students without representative sampling.

### 5.1 Recap of main descriptive findings

In this sample, short-form-video was used widely by participants. More than half of participants [67.7% (*n* = 268)] used Douyin for academic purposes, though recreational content was the main use. Most participants felt differently about the perceived educational benefits of the platform: most people felt that it was a learning platform and had self-reported negative experiences, such as difficulty controlling usage.

A descriptive discrepancy was identified between participants’ perceived educational value of short-video learning resources and their actual frequency of educational-oriented Douyin use. While many participants recognized the potential usefulness of learning-related short-video content, only a small share reported frequent learning-focused platform engagement.

Subgroup differences in perceived academic impact and self-regulatory behaviours were observed for the three sampled institutions. These differences are representative sample level patterns only. Explanations linking these patterns to self-regulatory capacity, academic socialization or academic pressure are tentative hypotheses for future work and are not tested directly in this data set.

Several participants reported habitual unconscious checking behaviour when using Douyin and self-reported difficulties in restricting usage time. Some participants reported fairly high daily short-video consumption time exceeding 3 h. This reported time metric reflects only self-reported consumption duration within this sample and must not be interpreted as diagnostic evidence of addiction or behavioural dependence.

### 5.2 Interpretations in conversation with existing literature

The descriptive patterns observed in this sample may be related to some previous publications. Participants reported accessing fragmented knowledge and examination preparation resources from short-video platforms. This was consistent with previous work suggesting microlearning opportunities of short-video media (Zhao, 2024; López-Carril et al., 2025). Similar to previous work (Montag et al., 2021), recreational content dominates user preferences even though educational content is also accessed by many respondents.

Many participants reported difficulty maintaining goal-oriented use and involuntary platform engagement. Self-reported behaviours could be consistent with neuropsychological results of existing literature. We collected no neurophysiological measurements and hence we can not directly test dopamine reward or executive function maturation hypotheses in this study. These ideas are based on external literature only for preliminary interpretation (Zhang and Huang, 2022; Kirschner and De Bruyckere, 2017).

Regarding perceived academic consequences, our sample had mixed positive negative subjective evaluations, echoing previous work suggesting that short-video related outcomes are highly context dependent rather than uniformly beneficial or harmful (Zhang, 2025). The difference between perceived educational value and actual learning oriented use observed in our sample is also related to general technology adoption research which states that positive perceived utility does not translate into consistent goal-directed behaviour (Sheeran and Webb, 2016). Since parallel validated items measuring formal behavioural intention were not included in our questionnaire, we avoid using the formal construct label “intention-behaviour gap” and only describe the observed empirical discrepancy.

### 5.3 Revisiting interpretive theoretical lenses

This paper relied on Social Cognitive Theory (Bandura, 1986). Sample level descriptive patterns of environmental cues, individual cognition and self-regulation are broadly correlated with reciprocal determinism of Social Cognitive Theory, however without valid multiitem scales measuring each theoretical latent variable, we cannot test these hypotheses.

Technology Acceptance Model (Davis, 1989) was used as background. We used no valid TAM measurement items so we cannot test TAM related hypotheses empirically. The difference between perceived value and real use can only be regarded as loosely suggestive and does not form part of the test of TAM.

Cognitive load theory and distraction conflict theory were used as interpretive lenses to analyze attention related problems. Intense/extra/germane cognitive load and validated attentional conflict constructs were not measured in our questionnaire. Our descriptive results thus cannot prove the mechanism assumptions proposed by these two theories.

### 5.4 Practical implications linked to sample findings

All practical implications below are derived strictly from descriptive patterns in the present sample and represent potential institutional responses rather than proven effective interventions. Further empirical evaluation would be required before confirming their real-world effectiveness.

Based on the sample observation that many undergraduates recognized short-video educational resources but struggled with self-regulation use behaviour, university educators and student affairs staff might consider developing targeted digital literacy training to help students distinguish high quality educational short-video content from fragmented entertainment content and build practical behaviours for short-video platform usage.

Given the descriptive subgroup differences observed across institutions (untested for statistical significance), university administrators from different-tier institutions may take differentiated perspectives when designing digital-literacy support. Tentatively, institutions whose students report more self-reported usage-regulation difficulties may prioritize practical behavioural-management modules within student support activities.

In addition, considering that educational short-video resources exist but are not frequently utilized for learning purposes in this sample, university teachers may appropriately incorporate high-quality short-video clips as supplementary out-of-class learning materials, to guide students toward goal-oriented use of short-form-video platforms.

### 5.5 Limitations reflected in discussion

Several important limitations should be kept in mind when interpreting these results. First, snowball convenience sampling limited to three Xuzhou based institutions. This restricts external validity and might lead to sampling bias. Second, all data are self-report responses. Self-report bias and common-method bias are unavoidable. Third, this cross-sectional design only produces descriptive associations. Causes can not be explained. Fourth, we did not use fully valid multi-dimensional scales for core constructs. We collected only single item self-regulatory behaviour indicators and no neurophysiological or objective academic performance measures were collected. None of mediation or mechanism hypotheses from external literature could be tested statistically in this dataset.

## 6 Conclusions and future research directions

### 6.1 Synthesis of principal findings

There are several conclusions of this study. Douyin has been widely adopted among students in China. Most of the students use it for academic purposes. However, recreational and entertainment use remains the main motivation for use, suggesting that instrumental learning applications are secondary drivers rather than primary drivers.

The relationship between Douyin use and perceived academic impact is bidirectional and context-dependent. Platform use does not result in uniformly directional effects, but produces simultaneous positive and negative effects that differ between users and implementation contexts. This paradoxically puts Douyin as a technological artifact with naturally ambivalent properties, capable of improving knowledge acquisition and cognitive efficiency while reducing sustained attention and temporal self-regulation.

The reasons for these perceived effects are self-reported. Beneficial effects are primarily professional knowledge development and faster access to information. Learners can supplement formal instruction with just-in-time resources, while negative effects are self-reported consumption patterns that consume study time and attention fragmentation. Study plan suspension is common among major minority populations, and such effects are often enough to warrant institutional attention.

Educational content on the platform may be beneficial for some participants, although self-reported experiences are highly individual and depend on content content quality and integration with structured learning objectives. Self-reports, usage patterns, unconscious activation during study episodes and self-regulatory-related behaviours are factors that participants correlated with the interaction with platform and self-perceived consequences. The main contribution of this work is descriptive self-report evidence of Chinese undergraduates’ perceived consequences for learning and self-regulatory behaviours for Douyin use in three different local institutions.

### 6.2 Limitations

There are several limitations of this paper that should be taken into account when considering its results. First, data were collected using snowball convenience sampling among students from 3 Xuzhou-based higher education institutions. Snowball sampling introduces potential sampling bias, and sample cannot be generalized to Chinese students nationwide; only three local institutions are covered. Second, data collected are self-reported questionnaire responses, which can lead to self-report bias, as well as common-method bias, since outcome and predictor data are collected from the same survey. Third, no fully validated multiitem standardized scales were used to represent core constructs. Only several single item questions were used to capture self-regulatory related behaviours, so formal reliability testing could not be performed. Fourth, the cross-sectional survey only yields descriptive patterns. It cannot establish causal relationships between variables, nor can it test mediation or moderation mechanism statistically. Thus, all observed associations should be treated as descriptive patterns in this sample rather than confirmed causal or moderated effects. Fifth, objective institutional academic-performance records such as GPA were not collected; all academic-related outcomes are based on participants’ subjective perceptions.

### 6.3 Practical recommendations

All suggestions listed below are potential institutional responses based solely on the descriptive patterns observed within this sample and are not proven-effective interventions. Further empirical evaluation is required prior to large-scale campus promotion and broad implementation across diverse student cohorts. None of these proposals constitute ready-to-deploy campus policy because our cross-sectional self-report dataset cannot establish causal links between proposed measures and improvements in undergraduates’ short-video-related learning experiences.

Because many participants reported difficulty controlling Douyin use in algorithm driven, highly immersive short video environment, university educators and student affairs staff could design digital literacy activities for undergraduates. Practical modules could cover personal usage limits, trigger situations for mindless scrolling, and distinguish entertainment-based content from knowledge-based content, helping students avoid habitual scrolling and self-management awareness in everyday studies. While many participants recognized the educational value of short videos, they reported rarely learning-oriented Douyin use in real life. Course instructors could curate high quality, discipline-relevant short videos as additional outof-class learning material. Such short videos can complement formal textbook content, expand informal learning pathways and gently guide students towards goaldriven shortform videos rather than aimless recreational scrolling. Nevertheless, educators must bear in mind that these clips function merely as auxiliary resources and cannot substitute for systematic teacher-led in-person teaching and formal curriculum delivery.

In addition, institutional practitioners should account for divergent student backgrounds when designing support measures. Perceived academic-related consequences of short-video use vary across participants from different institutional tiers; hence one-size-fits-all solutions ought to be avoided. Follow-up longitudinal or intervention-focused research is strongly encouraged to test whether these proposed practices yield measurable improvements for student learning and self-regulatory-related behaviours.

## Data Availability

The raw data supporting the conclusions of this article will be made available by the authors, without undue reservation.

## References

[ref1] Abdullah F. Ward R. (2016). Developing a general extended technology acceptance model for E-learning (GETAMEL) by analyzing commonly used external factors. Comput. Hum. Behav. 56, 238–256. doi: 10.1016/j.chb.2015.11.036

[ref2] Bandura A. (1986). Social Foundations of Thought and Action: A Social Cognitive Theory. Englewood Cliffs, NJ: Prentice-Hall.

[ref3] Baron R. S. (1986). Distraction-conflict theory: progress and problems. Adv. Exp. Soc. Psychol. 19, 1–40. doi: 10.1016/S0065-2601(08)60211-7

[ref4] Baumgartner S. E. Lemmens J. S. Weeda W. D. Huizinga M. (2017). Measuring media multitasking: development of a short media multitasking measure for adolescents. J. media Psychol. 29, 92–101. doi: 10.1027/1864-1105/a000167

[ref5] Chen J. (2025). A study on innovation in educational short videos from the perspective of new media. Q&A Learn. Guid. 9, 175–179.

[ref6] Cheng Y. Q. (2025). Development and effectiveness study of short video courses for improving foreign language proficiency based on the theory of “foreign language learning anxiety”. Writ. World. 26, 94–97.

[ref7] Davis F. D. (1989). Perceived usefulness, perceived ease of use, and user acceptance of information technology. MIS Q. 13, 319–340. doi: 10.2307/249008

[ref8] Gauvain M. (2010). Cognitive load theory. Psychol. Learn. Motiv. 55, 37–76. doi: 10.1007/978-1-4419-8126-4

[ref9] Gong Q. Tao T. (2024). The relationship between short video usage and academic achievement among elementary school students: the mediating effect of attention and the moderating effect of parental short video usage. PLoS One 19:e0309899. doi: 10.1371/journal.pone.0309899, 39585867 PMC11588245

[ref10] He Q. C. Jin X. M. (2019). Research on English learning models in mobile internet environments: the case of short video-based English learning to improve university English teaching. Campus Engl. 9, 2–3.

[ref11] He Y. R. Yang J. (2025). Short videos empowering international Chinese language education in Southeast Asia. West. Dev. 17, 187–189.

[ref12] Jonas Y. Majeed M. Alaaba B. D. (2024). “The effect of social media on academic performances of students in Ghanaian universities (a case of tamale technical university),” in Digital Transformation in the Customer Experience, (Palm Bay, FL: Apple Academic Press).

[ref14] Kirschner P. A. De Bruyckere P. (2017). The myths of the digital native and the multitasker. Teach. Teach. Educ. 67, 135–142. doi: 10.1016/j.tate.2017.06.001

[ref16] Lau W. W. F. (2017). Effects of social media usage and social media multitasking on the academic performance of university students. Comput. Hum. Behav. 68, 286–291. doi: 10.1016/j.chb.2016.11.043

[ref17] Li X. Li D. S. (2021). A study on the overseas dissemination strategies of the short-video platform ‘TikTok’: a case study of the United States, Japan, and Southeast Asia. J. Beijing Inst. Print. 29, 5–7.

[ref18] Lin W. X. (2023). Research on the impact of Douyin video scenarios on college students’ behavior in the social media era. Media Forum 6, 18–20.

[ref19] López-Carril S. Carpenter J. P. Watanabe N. M. (2025). TikTok in higher education: a learning tool to connect with industry and develop students’ digital skills. Innov. High. Educ. 51, 91–120. doi: 10.1007/s10755-025-09853-6

[ref21] Michikyan M. Subrahmanyam K. Dennis J. (2015). Facebook use and academic performance among college students: a mixed-methods study with a multi-ethnic sample. Comput. Hum. Behav. 45, 265–272. doi: 10.1016/j.chb.2014.12.033

[ref22] Montag C. Yang H. B. Elhai J. D. (2021). On the psychology of TikTok use: a first glimpse from empirical findings. Front. Public Health 9:641673. doi: 10.3389/fpubh.2021.641673, 33816425 PMC8010681

[ref25] Scherer R. Siddiq F. Tondeur J. (2019). The technology acceptance model (TAM): a meta-analytic structural equation modeling approach to explaining teachers' adoption of digital technology in education. Comput. Educ. 128, 13–35. doi: 10.1016/j.compedu.2018.09.009

[ref26] Sheeran P. Webb T. L. (2016). The intention–behavior gap. Soc. Personal. Psychol. Compass 10, 503–518. doi: 10.1111/spc3.12265

[ref27] Shi Y. T. (2023). The impact of short video platforms on college students’ ideological and political education and countermeasures. Ind. Technol. Voc. Educ. 21, 96–98.

[ref28] Shibchurn J. Yan X. (2015). Information disclosure on social networking sites: an intrinsic–extrinsic motivation perspective. Comput. Hum. Behav. 44, 103–117.

[ref29] Shutsko A. (2020). “User-generated short video content in social media: a case study of TikTok,” in Social Computing and Social Media, ed. Meiselwitz G. (Cham: Springer).

[ref30] Sweller J. (1988). Cognitive load during problem solving: effects on learning. Cogn. Sci. 12, 257–285. doi: 10.1207/s15516709cog1202_4

[ref31] Tang Y. Z. Yin F. Du G. (2025). Design and practice exploration of mathematical analysis visualization teaching cases based on multimodal representation and cognitive load theory. Educ. Progress 15, 831–840.

[ref32] Wang X. C. Tian F. R. (2025). The relationship between college students’ short-video addiction and academic performance: the mediating role of learning engagement and the moderating role of academic self-efficacy. J South China Norm Univ 1, 120–130.

[ref33] Wang H. F. Zhao S. M. (2025). The impact of Douyin app on college students and educational countermeasures from counselors’ perspective: a case of students at Shanxi Institute of Engineering Technology. Fujian Light Ind. Textiles 9, 69–73.

[ref34] Wei X. Z. (2022). The impact of short video platforms like Douyin on college students’ academic and daily lives. Electron. Compon. Inf. Technol. 6, 82–84.

[ref36] Zhang C. (2025). A study on enhancing the dissemination effectiveness of educational short videos. J Hubei Norm Univ 45, 151–156.

[ref37] Zhang J. Huang Q. Q. (2022). Research on challenges and countermeasures in building short video teaching resource platforms in higher education institutions. Mod. Inf. Technol. 6, 195–198.

[ref38] Zhang P. Yu J. X. Zhu K. (2023). A study on the relationship among short video use, parental intervention, and children’s academic performance. Journalism 5, 80–93.

[ref39] Zhao H. X. (2024). Research on vocational college students’ TikTok usage tendencies and guidance strategies in the context of internet plus. Voc. Educ. Dev. 13:2028.

[ref40] Zhao Y. Pinto Llorente A. M. Sánchez Gómez M. C. (2021). Digital competence in higher education research: a systematic review. Comput. Educ. 168:12. 10.1016/j.compedu.2021.104212 PMC9759745 36568577

[ref41] Zhou X. (2023). Current status, psychological mechanisms, and intervention strategies for college students’ short video dependency. Adv. Psychol. 13, –6034.

